# Acceptance and Use of Telepsychology From the Clients’ Perspective: Questionnaire Study to Document Perceived Advantages and Barriers

**DOI:** 10.2196/22199

**Published:** 2021-10-15

**Authors:** Beatriz Sora, Rubén Nieto, Adrian Montesano del Campo, Manuel Armayones

**Affiliations:** 1 Department of Psychology Rovira i Virgili University Tarragona Spain; 2 Department of Psychology eHealth Center, Open University of Catalonia Barcelona Spain; 3 eHealth Center Open University of Catalonia Barcelona Spain

**Keywords:** telepsychology, telepsychology advantages, telepsychology barriers, telepsychology use, telepsychology usefulness, intention to use telepsychology

## Abstract

**Background:**

Telepsychology is increasingly being incorporated in clinical practice, being offered in many psychotherapy centers, especially after the impact of the pandemic. However, there seems to be a remarkable discrepancy between the offer, or interest in, and real-world uptake of e-mental health interventions among the population. A critical precondition is clients’ willingness to accept and use telepsychology, although this issue has thus far been overlooked in research.

**Objective:**

The aim of this study was to examine people’s acceptance and use of telepsychology by adopting an extended model of the unified theory of acceptance and use of technology (UTAUT) that integrates perceived telepsychology advantages and barriers, usefulness perceptions, behavioral intention, and telepsychology use.

**Methods:**

An online survey was conducted with a convenience sample of 514 participants. Structural equation models were computed to test a mediation model.

**Results:**

Results supported the UTAUT model to explain participants’ acceptance and use of telepsychology. They showed a causal chain in which perceived telepsychology advantages and barriers were related to telepsychology use through the perceived usefulness of and intention to use telepsychology.

**Conclusions:**

Telepsychology use may be explained according to the UTAUT model when coupled with participants’ perceptions of telepsychology advantages and barriers. Mental health stakeholders could consider these factors in order to increase the acceptance and use of telepsychology.

## Introduction

### Background

Every year, a high percentage of the population requires mental health services [1]. However, not all people have adequate access to the specialized mental health care they need. Figures illustrating this vary widely between studies and depend on the definition given to mental health care. For example, a representative European sample evidenced that while 6.5% of people had a need for mental health care, more than 3% of them did not receive the appropriate treatment [2]. The negative consequences of failing to treat these problems are well documented in the literature and include poor health outcomes, suicide, divorce, substance abuse, child neglect and abuse, and youth delinquency [3-5]. Thus, finding solutions that spread access to mental health care throughout the population is critical.

Information and communication technologies (ICT) have great potential to facilitate access to interventions. In this regard, telepsychology, which the American Psychological Association (APA) defines as “the provision of psychological services using telecommunication technologies,” has appeared in recent years as an alternative to traditional face-to-face interventions, at least for a significant proportion of the population. Telepsychology involves the use of different electronic tools to deliver health care, which may range from telephones and fiber optics to interactive satellite video [6]. This work focuses on videoconferencing technology, which synchronously overcomes geographical barriers, thereby enabling people to see and talk to each other as if they were in the same room despite being apart.

Literature on telepsychology use (especially on the use of videoconferencing technology) has increased exponentially in recent years [7]. In this line, institutions such as the APA have created guidelines for the use of telepsychology [8]. Systematic reviews showing the positive effects of telepsychology have also appeared. For example, Varker et al [9] reviewed published research about the use of synchronous telepsychology to treat anxiety, post-traumatic stress disorder, and adjustment disorder. They found strong evidence pointing to the high-quality nature of this option, as well as to the equivalence between telephone- or videoconference-delivered interventions and face-to-face interventions. Although more research is needed, in general terms, available results suggest that telepsychology could produce equal results when compared to traditional interventions and that therapeutic alliance can be as successfully established in videoconference psychotherapy as in face-to-face interventions [10-12]. In addition, telepsychology is also gaining representativeness in routine clinical practice, especially after the pandemic. For instance, Pierce et al [13] surveyed a national sample of 2619 licensed psychologists in the United States and found that those practicing in outpatient facilities reported a 26-fold increase in telepsychology in response to the pandemic. Moreover, participants stated that 34.96% of their clinical work would be conducted via telepsychology after the pandemic ceases, reflecting an important shift in attitudes toward the use of telepsychology.

For really potentiating the use of telepsychology, a fundamental precondition, as with the implementation of any other new technology or application [14,15], is to study users’ willingness to accept and use it. In general, there seems to be a remarkable discrepancy between the interest in and real-world uptake of e-mental health interventions among the population [16,17]. Studies have shed light on the fact that willingness to participate in e-mental health interventions is limited, either because of a low uptake rate among patients or low acceptance by the population in general [18-21].

Unfortunately, research has overlooked this issue. Only 3% of studies on eHealth, in general, focus on people’s acceptance, making this an understudied domain [22,23]. Consequently, there is limited knowledge about people’s genuine attitudes towards e-mental health and the reasons behind their intention to use it [14]. A comprehensive understanding of determinant factors for acceptance and use of e-mental health, in general, and telepsychology, in particular, represents an essential first step towards creating successful telepsychology services. This is a pressing issue in the current context of social distancing and the telepsychology revolution [13].

### Acceptance and Use of Telepsychology: Unified Theory of Acceptance and Use of Technology (UTAUT) Model

Technology acceptance is a relatively mature area of research, and there is a significant amount of literature on the matter [24]. It presents several models, based mainly on social psychology, to explain people’s acceptance and use of new technologies. Some more widely accepted theories on the use behavior of new technologies are the technology acceptance model (TAM) [25], theory of planned behavior (TPB) [26], theory of reasoned action (TRA) [27], motivational model (MM) [28], combined TAM and TPB (C-TAM-TPB) [29], model of personal computer use, theory of innovation diffusion (TID) [30], and social cognitive theory (SCT) [31]. These theories and models have since been fused to create a more complex framework: the unified theory of acceptance and use of technology (UTAUT) [24]. This model was proposed in order to combine the contributions of the mature yet fragmented literature on technology acceptance and to establish a unified theory to explain individuals’ use and acceptance of technology. The UTAUT contemplates 4 core determinants of use and intention: (1) performance expectancy, (2) effort expectancy, (3) social influence, and (4) facilitating conditions. In this respect, Koivumäki et al [22] summarized their definitions as follows. Performance expectancy reflects the degree to which using a technology will facilitate the achievement of some goal (ie, technology will enhance quality of life performance). It involves determinants such as perceived usefulness, extrinsic motivation, job fit, relative advantages, and outcome expectations from technology acceptance studies. Effort expectancy represents the degree of ease associated with the use of a technology, such as ease of use and its determinants and complexity. Social influence is defined as the extent to which it is perceived that significant others (eg, family or friends) believe that they should use a technology. It reflects the determinants of social factors, subjective norms, and image from the technology acceptance literature. Facilitating conditions represent perceptions of the external resources and infrastructure that support the use of an information and technology system (eg, perceived behavioral control and compatibility). Finally, behavioral intention was defined as a measure of the strength of one’s intention to perform a specific behavior [26]. It reflects the acceptance to use eHealth tools. More specifically, the UTAUT model [24] proposes that performance expectancy, effort expectancy, and social influence are direct predictors of the intention to use an innovative technology and that facilitating conditions and behavioral intention are direct determinants of actual use.

This model has been applied and tested in multiple contexts to provide insight into the forces that motivate individuals to adopt technology. In the case of eHealth, most empirical research singles out performance expectancy (eg, perceived usefulness) as the strongest predictor of technology acceptance [32-37]. Perceived usefulness is defined as the extent to which a person believes that using a system will help him or her to achieve their objectives [25]. It essentially captures people’s cognitive expectations about the performance of the system, which determines the intention of technology use. In other words, if people believe that the new technology, in our case telepsychology, can help them, they will present higher intention to use it compared to those who do not perceive any benefit. In this line, several meta-analyses in the eHealth field show that perceived usefulness has the largest effects on behavioral intention (eg, [38,39]). Likewise, behavioral intention is the main predictor of use behavior (eg, [32,40]). Unfortunately, as mentioned previously, we are not aware of any study that has specifically examined telepsychology; accordingly, people’s acceptance and use of psychotherapy through videoconferencing — that is, telepsychology — are still unknown.

### Additional Determinants of Telepsychology Acceptance and Use: Advantages and Barriers

The UTAUT model [24] underpins the determinants of technology acceptance and use, making it the most complete model for predicting technology acceptance and use. However, given the complex nature of eHealth acceptance and its determinants, it necessary to extend this model and adapt it to different contexts [14].

A relevant line of research has expanded the UTAUT model by including success factors (eg, advantages) and resistance factors (eg, barriers) that drive people to adopt and use a certain technology (eg, [14,36,41,42]). At the initial stage of adoption of a new ICT, people have limited knowledge and thus struggle to decide whether to use it. There are likely opportunity factors that motivate them to use the new technology, as well as barrier or risk factors (understood as perceptions and not only as actual obstacles) that cause them to hesitate using it. Hence, perceived advantages and barriers represent reasons for or against the use of a technology [43].

Literature on eHealth suggests that the inclusion of ICT in mental health care services may pose several advantages and barriers for patients that conventional face-to-face interventions do not. Ebert et al [41] summarized them as follows. Advantages include the fact that (1) e-mental health interventions are more easily accessible at any time and place, (2) e-mental health interventions facilitate the integration of acquired skills in daily life because of the patients' active roles, (3) participants can work at their own pace and go through materials as often as they want, (4) travel time and costs are removed, and (5) e-mental health interventions may attract people who do not make use of traditional mental health services. The following barriers have been pointed out: (1) low expectancies regarding its effectiveness, (2) reservations regarding data security, (3) low comfort using such programs, (4) influence by important social contacts (eg, family and health professionals), (5) negative attitudes towards seeking psychological help in general, (6) low internet experience, and (7) high internet anxiety. Further studies added low internet orientation in health problems and insufficient knowledge of eHealth interventions [21] as well as worries about impersonal interaction [44].

In sum, research has paid special attention to advantages and barriers that may determine e-mental health care services. However, authors such as Henneman et al [14] call for further research, as knowledge about eHealth adoption barriers and advantages remains limited. For example, only a handful of empirical studies have simultaneously examined advantages and barriers [42], indicating that, to facilitate the use of eHealth applications, they need to integrate ease of use and usefulness with a certain level of reliability. Finally, in terms of telepsychology, we are not aware of any study that has specifically focused on psychotherapy through videoconferencing technology. Thus, the advantages of and barriers to adopting telepsychology remain to be studied.

### Research Purpose and Hypotheses

**Figure 1:**
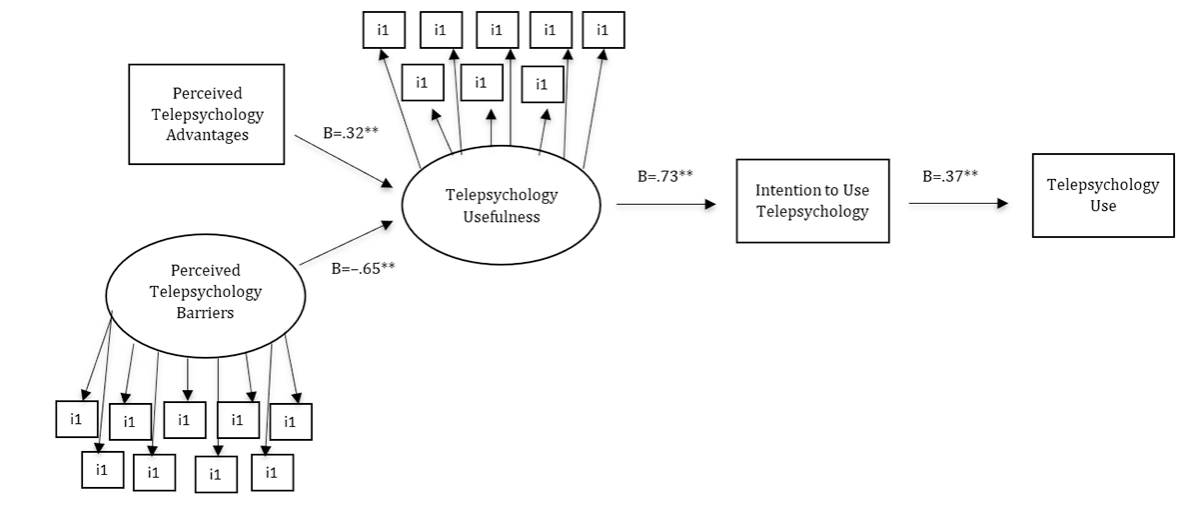
Results of the structured equation model (SEM) for the hypothesized unified theory of acceptance and use of technology (UTAUT) model applied to telepsychology. All parameters are standardized; results are controlled for sex and age; telepsychology use is a dummy variable: (1) no and (2) yes. *P<.05, **P<.01.

This study aimed to obtain a deeper understanding of people’s acceptance and use of telepsychology by examining the determinant factors according to an extended UTAUT model that includes perceived telepsychology advantages and barriers. It represents a first step in the study of telepsychology acceptance and use. Thus, our goal was to examine the strongest predictors of technology use in the UTAUT model following eHealth literature (ie, usefulness perceptions and behavioral intention). Our conceptual model is displayed in Figure 1 and integrates telepsychology advantages and barriers as antecedents of usefulness perceptions and behavioral intention. It also considers usefulness perceptions and behavioral intention as key mediating mechanisms for telepsychology use. So, first, this model proposed that perceived telepsychology advantages and barriers may determine telepsychology usefulness. The more advantages and fewer barriers that are perceived, the more useful telepsychology will be perceived. Second, telepsychology usefulness is related to telepsychology use because, when people believe that telepsychology can help them, that is, it is useful, then they will have a higher intention to use it. Third, intention to use is related to use. Intention to use is a natural predictor of technology use. Finally, note that in the UTAUT model, sex and age play a moderator role in the relationship between usefulness and behavioral intention. They have significant effects in the model, so we included them as control variables to take into account their effects on telepsychology acceptance and use. Accordingly, we had the following hypotheses:

- Hypothesis 1 stated that perceived telepsychology barriers are negatively related with telepsychology usefulness.
- Hypothesis 2 stated that perceived telepsychology advantages are positively related with telepsychology usefulness.
- Hypothesis 3 stated that telepsychology usefulness is positively related with the intention to use telepsychology.
- Hypothesis 4 stated that the intention to use telepsychology is positively related to telepsychology use.
- Hypothesis 5 stated that telepsychology usefulness mediates the relationship between perceived telepsychology barriers (H5a) and advantages (H5b) and the intention to use telepsychology.
- Hypothesis 6 stated that the intention to use telepsychology mediates the relationship between telepsychology usefulness and telepsychology use.

## Methods

### Procedure and Sample

As we wanted to study the general population perspective, we recruited a convenience sample through an online advertisement published on our university’s website. The ad explained the research project, explained its main objective, and asked for volunteers who might be willing to participate in our research by taking an open online survey. The ad also provided the link to the survey, which was implemented using the Qualtrics platform. In order to increase response rates, the researchers sent this link along with a brief summary of the research project to their contacts via email.

All of the surveys implemented in the host institution for research purposes are implemented using the Qualtrics platform, since it guarantees data protection. Qualtrics allows downloading responses in different formats. Once the survey closed, we downloaded data in Excel format and moved it to SPSS.

The survey was responsive to different devices, but we recommended that potential participants complete it using a computer since it was perceived by the research team and users who tested it in advance to be easier. The survey assessed the dimensions (presented in the order used for the survey) that are presented in the following sections. Questions had to be completed to progress in the survey and move to the next screen (if a question was not answered, the system provided an error message). There was a maximum of 10 screens (some of them did not appear if they were not applicable for the specific participant by taking into account his or her previous responses). There was not a specific number of items per screen since it depended on the type of item, but we always tried to avoid excessive scrolling.

The user’s IP was not registered to guarantee anonymity; however, the Qualtrics system maintains an opened survey and saves a participant’s progress for a week. So, during this period, if participants stopped and restarted the survey, they were directed to the exact place they were when they left the survey (if they used the same computer and browser). At the bottom of the screen, there was a progress bar.

The only inclusion criterion for participation was being older than 18 years. In the data collection process, anonymity and confidentiality were guaranteed, and participants provided their consent to participate by accessing the survey and accepting the conditions (ie, all responses were anonymous, no personal data were gathered, and participants could stop participating at any time). No incentive was offered to participants. The protocol was previously approved by the university’s ethics committee. The final sample was composed of 514 participants. A total of 568 persons entered the system; of these, 54 did not complete the survey and were excluded.

### Measures

The current literature did not offer measures for the specific variables in this study. Accordingly, a specific online survey was created following similar studies and taking into account the available literature. The survey was created and reviewed in an iterative manner by the authors. In addition, before making the survey available to participants, it was tested by 4 volunteers who suggested changes that were implemented. They could judge both the format and functionality of the online survey and the content of the items. Regarding the content of items, they could assess if they were appropriate for the targeted construct and easily understandable. The measures of this study were perceived telepsychology advantages, perceived telepsychology barriers, telepsychology usefulness, intention to use telepsychology, and telepsychology use.

#### Perceived Telepsychology Advantages

The perceived telepsychology advantages were assessed by computing the participants’ answers to the statement: “Please indicate the different advantages that might motivate you to use telepsychology.” According to the literature (eg, [41]), the possible answer options were: (1) lower economic cost, (2) the possibility of receiving treatment from home, (3) access to specialized treatment, (4) greater anonymity, (5) as a complement to face-to-face psychotherapy, and (6) none of the above. All “yes” responses were given a value of 1, except for the last option (none of the above), which was given a value of 0. The sum of the marked (“yes”) options was the index that represented perceived telepsychology advantages.

#### Perceived Telepsychology Barriers

The perceived telepsychology barriers were measured by means of a self-developed scale. It included a general statement: “Please indicate to what extent the following elements would present a barrier to doing online psychotherapy,” with 9 items that reflected the main barriers identified in the literature (eg, [19,41,43]). These items were: (1) it would prevent me from having close or warm contact with my therapist, (2) it would prevent me from expressing my emotions or feelings, (3) I would not be able to pick up on the therapist’s nonverbal language well, (4) the therapist would not understand my nonverbal language well, (5) there would be online confidentiality risks, (6) I would not have enough connection speed or the connection would cut out, (7) there is scarce scientific evidence for the efficacy of telepsychology, (8) there is scarce legal regulation, and (9) I lack the knowledge or resources required to videoconference. The response options varied from 1 (not at all) to 5 (very much so). The mean of the items was the index that represented perceived telepsychology barriers.

#### Telepsychology Usefulness

Telepsychology usefulness was also measured using a self-developed, 5-point Likert scale. It included the general statement: “Please indicate to what extent you think telepsychology can be effective for the following issues,” with 8 items reflecting the most common presenting problems in psychotherapy. More specifically, the items were: (1) improvement of mood disorders (eg, depression, anxiety), (2) improvement of relational problems (eg, couple or family problems), (3) improvement of work-related stress problems, (4) health problems (eg, chronic pain, diet, fibromyalgia), (5) personal growth issues, (6) mild psychological problems (interfering little with daily life), (7) moderate psychological problems (interfering moderately with daily life), and (8) severe psychological problems (interfering seriously with daily life). The response options ranged from 1 (not at all) to 5 (very much so).

#### Intention to Use Telepsychology

Intention to use telepsychology was assessed with a mono-item scale asking: “If you had a problem today, how likely would you be to use telepsychology?” The response options ranged from 1 (very unlikely) to 5 (very likely).

#### Telepsychology Use

Telepsychology use was measured as a dummy variable with the following question: “Have you ever attended any kind of online psychological therapy?” Two answer options were provided: (1) no and (2) yes.

### Analysis

The following preliminary analyses were computed: mean, SD, and correlation. In addition, given that the measures of telepsychology barriers and usefulness were self-developed, we examined their validity and reliability through confirmatory factor analysis (CFA) and Cronbach alpha. Later, structural equation models (SEM) were performed to test our hypotheses on mediation effects. Two models were computed: (1) a full model that included the direct and indirect relationships among all our variables and (2) a hypothesized UTAUT model. Mplus software [43] was used. Maximum likelihood was employed to estimate the parameters of the model. Model adjustment was assessed through chi-squared statistics and fit indices, such as the Tucker-Lewis index (TLI), root mean square error of approximation (RMSEA), and weighted root mean square residual (WRMR). A good fit was defined as values higher than .90 for TLI, values lower than .08 for RMSEA, and values lower than 1 for WRMR [45,46].

## Results

### Sample Characteristics

Of the 514 participants, 79.8% (410/514) were women, and 20.2% (104/514) were men. The mean age was 36.27 (SD 10.35) years. Only 0.4% (2/514) of the participants had not completed any level of education, while 2.7% (14/514) had studied at elementary school, 27.0% (139/514) had studied at secondary school, 43.2% (222/514) had studied at college, and 26.7% (137/514) had studied a postgraduate course. Up to 61.9% (318/514) of participants reported having undergone face-to-face psychotherapy, and 6.4% (33/514) had experienced telepsychology formats. Finally, 17.1% (88/514) had a monthly salary lower than €600 (US $708.54), 16.7% (86/514) had a salary between €600 and €999 (US $1179.71), 26.7% (137/514) earned between €1000 (US $1180.86) and €1499 (US $1770.14), 20.6% (106/514) earned between €1500 (US $1771.32) and €1,999 (US $2360.58), 10.7% (55/514) had an annual income between €2000 (US $2361.72) and €3000 (US $3542.63), 3.5% (18/514) earned more than €3000, and finally 24 participants did not disclose their salary range.

### Preliminary Analysis

Table 1 shows our descriptive results. Most of the variables were significantly correlated with the others. Noteworthy is the high correlation between telepsychology usefulness and intention to use telepsychology (*r*=0.50). Table 2 presents our confirmatory factor analysis. Goodness-adjustment indexes pointed out an appropriate adjustment of data to model for telepsychology barriers and usefulness measures (see [46-48]). Cronbach alphas were .83 for telepsychology barriers and .92 for telepsychology usefulness. Thus, it is possible to conclude that the validity and reliability of these scales were appropriate.

**Table 1:** Descriptive analysis and correlations.

Variable			Sex		Age^a^		Perceived telepsychology advantages^b^		Perceived telepsychology barriers^c^		Telepsychology usefulness^d^		Intention to use telepsychology^e^		Telepsychology use^f^
**Sex**															
	*r*	1		–0.03		0.03		0.09		0.01		0.05		–0.03	
	*P* value	—^g^		.55		.54		.06		.76		.26		.45	
**Age**															
	*r*	–0.03		1		–0.10		–0.02		–0.22		–0.03		0.01	
	*P* value	.55		—		.02		.71		<.001		.48		.87	
**Perceived telepsychology advantages**															
	*r*	0.03		–0.10		1		–0.08		0.37		0.32		–0.04	
	*P* value	.54		.02		—		.09		<.001		<.001		.36	
**Perceived telepsychology barriers**															
	*r*	0.09		–0.02		–0.08		1		–0.38		–0.25		0.11	
	*P* value	.06		.71		.09		—		<.001		<.001		.01	
**Telepsychology usefulness**															
	*r*	0.01		–0.22		0.37		–0.38		1		0.50		–0.11	
	*P* value	.76		.00		<.001		<.001		—		<.001		.01	
**Intention to use telepsychology**															
	*r*	0.05		–0.03		0.32		–0.25		0.50		1		–0.18	
	*P* value	.26		.48		<.001		<.001		<.001		—		<.001	
**Telepsychology use**															
	*r*	–0.03		0.01		–0.04		0.11		–0.11		–0.18		1	
	*P* value	.45		.87		.36		.01		.01		<.001		—	

^a^Mean (SD): 36.27 (10.35) years.

^b^Mean (SD): 2.61 (1.28).

^c^Mean (SD): 3.04 (1.23).

^d^Mean (SD): 3.14 (1.14).

^e^Mean (SD): 2.69 (1.28).

^f^Dummy variable: (1) yes and (2) no.

^g^Not applicable.

**Table 2:** Fit indices for the structural equation model.

Variables	χ²	*df*	*P* value	χ ²/*df*	TLI^a^	RMSEA^b^	WRMR^c^
CFA^d^: perceived telepsychology barriers	75.16	18	<.001	4.17	.93	.08	N/A^e^
CFA: telepsychology usefulness	72.20	16	<.001	4.51	.96	.08	N/A
Full model: direct and indirect effects	291.95	10	<.001	29.19	.89	.08	.91
UTAUT^f^ model	291.95	10	<.001	29.19	.90	.08	.95

^a^TLI: Tucker-Lewis index.

^b^RMSEA: root mean square error of approximation.

^c^WRMR: weighted root mean square residual.

^d^CFA: confirmatory factor analysis.

^e^N/A: not applicable.

^f^UTAUT: unified theory of acceptance and use of technology.

### Hypothesis Testing

Table 2 presents the SEM results. Both the full and UTAUT models indicated an acceptable fit because the adjustment indexes were very similar. However, taking into account the theoretical framework and the fact that the UTAUT model presented a slightly better adjustment compared to the full model, we adopted the UTAUT model results.

Figure 1 displays the model results and supports all hypotheses. Hypothesis 1 was supported, as there was a significant negative relationship between perceived telepsychology barriers and telepsychology usefulness. In other words, the higher the perceived barriers to telepsychology were, the less useful participants perceived it to be. Hypothesis 2 was also supported: Perceived telepsychology advantages were positively related with telepsychology usefulness. This means that the greater the perceived advantages of telepsychology, the more useful participants perceived it to be and the higher their intention of use was. Note that the effect of perceived telepsychology barriers on telepsychology usefulness was stronger than the association between perceived telepsychology advantages and telepsychology usefulness.

Hypothesis 3, which suggested a positive relationship between telepsychology usefulness and the intention to use telepsychology, was supported as well, with results showing a significant positive relationship. In other words, participants that perceived telepsychology as useful tended to show a greater intention to use it.

Hypothesis 4 was supported, as there was a significant positive relationship between the intention to use telepsychology and actual telepsychology use, indicating that participants with higher levels of intention to use telepsychology presented higher telepsychology use than those with low intention.

Finally, all the hypotheses about mediation effects were also supported. Regarding the mediator role of telepsychology usefulness (H5), the results showed that it mediated the relationship between perceived telepsychology advantages (B=.23, *P*=.00) and perceived telepsychology barriers (B=–.47, *P*=.00) and the intention to use telepsychology.

The results presented a significant indirect effect of telepsychology usefulness on telepsychology use through the intention to use telepsychology (H6), showing that the intention to use telepsychology mediates the relationship between telepsychology usefulness and telepsychology use (B=.27, *P*=.00). In sum, perceived advantages and barriers affected participants telepsychology use through their perception of telepsychology usefulness and their intention to use telepsychology.

## Discussion

### Principal Findings

This study is one of few to examine the acceptance and use of telepsychology from participants’ perspectives. It draws from the UTAUT model to explain how people accept and use telepsychology, taking into account not only UTAUT factors (usefulness) but also additional determinants such as perceived telepsychology advantages and barriers.

Our results supported the viability of the UTAUT model in assessing telepsychology acceptance and use. It showed that telepsychology use is predicted by telepsychology usefulness and the intention to use telepsychology. These results are congruent with the extensive literature on the acceptance and use of new technology and on eHealth acceptance and use in particular [22,35,36,38-40].

Perceived advantages and barriers also played a relevant role in explaining telepsychology acceptance and use. These factors determined participants’ perceptions of telepsychology usefulness, which affected their intention to use it and, in turn, their actual use of it. A positive perception in the balance between telepsychology advantages and barriers seems to be critical in determining whether people will accept and use this treatment option, with barriers having the strongest effect. These results are also congruent with previous literature on perceived eHealth advantages and barriers [14,36,41,42]. In this respect, it is worthy to mention that this literature has pointed out a discrepancy between low performance expectancy and actual efficacy of eHealth interventions [14,49]. This discrepancy is at least partially supported by our study, as it illustrates the critical role of perceived barriers in explaining telepsychology usefulness. Therefore, the need for further and transparent information and education to clarify misconceptions, especially those related to telepsychology barriers, was clear.

Overcoming barriers and fostering a positive perception of telepsychology has become a central issue since the outbreak of the COVID-19 pandemic. In this new context of social distancing, online psychotherapy has become more a necessity than an option. Many European and American mental health providers and policies relied on using technology to mitigate COVID-19 risks and to respond to elevated mental health demands. Our results can help stakeholders to strategically design ways of facilitating access and readiness to this treatment modality by focusing on the tested UTAUT model. Furthermore, as suggested by Pierce et al [13], telepsychology has come to stay, beyond the response to the pandemic crisis, and, therefore, the maturity of the field needs accelerated development to equal its expected widespread dissemination in routine practice.

Finally, the focus of this study was on the perception of synchronous videoconferencing, which is the most similar form of internet-delivered treatment to face-to-face psychotherapy. Other forms of telepsychology, such as internet-based treatment or self-guided, internet-based psychological interventions, could share some critical aspects with the model presented in our study. However, further research should be carried out to understand specific barriers and perceived usefulness when the intervention involves minimal or nonexistent contact with professionals.

### Limitations

Despite the interesting insight provided by this study, some limitations must be taken into consideration. First, all the measures were self-reported by participants, making common method variance possible. Future research should consider using additional measures from other sources. Second, the research design of this study was cross-sectional. Thus, it was not possible to infer causal relationships. Further research with longitudinal designs will be necessary to appropriately examine the possible causal effects as well as the stability of the UTAUT model over time.

Third, a convenience sampling method was used to collect data, which may limit the extrapolation of our results, especially to clinical settings. However, as it happens in other studies [50], it is unlikely to jeopardize the validity of our results, and it seems more probable that our results would be similar in other samples. In addition, since more than 60% of participants had used psychotherapy services in the course of their life, restrictions to the generalizability of the results to actual patients are lessened. In any case, further replication studies are needed. Fourth, 79.8% of the sample were women. This composition could have influenced our results, and it can make extrapolating them to a male sample difficult. Nevertheless, psychotherapy services are also more commonly used by women than men. Further research is needed to replicate and validate our results.

### Future Research

This study represents a first step towards applying the UTAUT model to telepsychology. However, we focused on the most important factors to explain participants’ acceptance and use of telepsychology, thereby overlooking other factors that are also relevant. In fact, it is congruent with the recent work by Ammenwerth [51], who concluded that the acceptance of a technology depends on multiple additional factors that has been overlooked, such as socio-organizational, workflow, cultural, or emotional aspects as well as differences in user groups (physicians, nurses, patients). For example, a critical personal determinant in telepsychology acceptance and use could be a previous mental health diagnosis or treatment. Thus, future research is needed to examine these additional factors to gain a deeper understanding of telepsychology acceptance and use. Such factors could be ease of use, facilitators, or moderator variables. Telepsychology is a new field of study that requires further research, especially from the users’ perspectives. A promising line of patient-focused research consists of involving users in the development of tools and platforms used to deliver interventions in order to meet their needs and minimize perceived barriers. Optimizing the engagement of participants in interventions is a key aspect for achieving successful treatment outcomes. Finally, it is probable that consumers’ and professionals’ perceptions about online psychotherapy had shifted as they have been impelled to experience the setting due to the pandemic crisis. Data collection was carried out before the outbreak, and, therefore, we could not take into account the social context when developing the UTAUT model for telepsychology. It is probable that society’s perception about telepsychology has changed. Hence, additional research is necessary to better understand telepsychology acceptance by society.

### Practical Implications

This study describes the main factors that must be taken into account to promote acceptance and use of telepsychology among potential clients. Our results provide evidence of the need to foster a positive perception of telepsychology, with a focus on its advantages, and to come up with ways to overcome perceived barriers that do not otherwise hinder conventional face-to-face psychotherapy. In this respect, mental health care stakeholders have a critical role, as van Voorhees et al demonstrated [52], showing that uptake of an e-mental health intervention increased when clinicians adopted a focus on client-centered information aimed at intrinsic motivation.

## Abbreviations

APA: American Psychological Association
C-TAM-TPB: combined technology acceptance model and theory of planned behavior
CFA: confirmatory factor analysis
ICT: information and communication technology
RMSEA: root mean square error of approximation
SCT: social cognitive theory
SEM: structural equation models
TAM: technology acceptance model
TID: theory of innovation diffusion
TLI: Tucker-Lewis index
TPB: theory of planned behavior
TRA: theory of reasoned action
UTAUT: unified theory of acceptance and use of technology
WRMR: weighted root mean square residual

## References

[1] World Health Organization (2001). The World Health Report.

[2] Alonso J, Codony M, Kovess V, Angermeyer MC, Katz SJ, Haro JM, De Girolamo G, De Graaf R, Demyttenaere K, Vilagut G, Almansa J, Lépine JP, Brugha TS (2007). Population level of unmet need for mental healthcare in Europe. Br J Psychiatry.

[3] Blumenthal SJ, Kagen J (2002). The Effects of Socioeconomic Status on Health in Rural and Urban America. JAMA.

[4] Johnson DR, Booth A (1990). Rural Economic Decline and Marital Quality: A Panel Study of Farm Marriages. Family Relations.

[5] Kim KJ, Conger RD, Lorenz FO, Elder GH (2001). Parent–adolescent reciprocity in negative affect and its relation to early adult social development. Developmental Psychology.

[6] Rees CS, Haythornthwaite S (2007). Telepsychology and videoconferencing: Issues, opportunities and guidelines for psychologists. Australian Psychologist.

[7] Simpson S (2009). Psychotherapy via videoconferencing: a review. British Journal of Guidance & Counselling.

[8] Guidelines for the Practice of Telepsychology. American Psychological Association.

[9] Varker T, Brand RM, Ward J, Terhaag S, Phelps A (2019). Efficacy of synchronous telepsychology interventions for people with anxiety, depression, posttraumatic stress disorder, and adjustment disorder: A rapid evidence assessment. Psychol Serv.

[10] Sucala M, Schnur JB, Constantino MJ, Miller SJ, Brackman EH, Montgomery GH (2012). The therapeutic relationship in e-therapy for mental health: a systematic review. J Med Internet Res.

[11] Flückiger C, Del Re AC, Wampold BE, Horvath AO (2018). The alliance in adult psychotherapy: A meta-analytic synthesis. Psychotherapy (Chic).

[12] Simpson SG, Reid CL (2014). Therapeutic alliance in videoconferencing psychotherapy: a review. Aust J Rural Health.

[13] Pierce BS, Perrin PB, Tyler CM, McKee GB, Watson JD (2021). The COVID-19 telepsychology revolution: A national study of pandemic-based changes in U.S. mental health care delivery. Am Psychol.

[14] Hennemann S, Beutel ME, Zwerenz R (2016). Drivers and Barriers to Acceptance of Web-Based Aftercare of Patients in Inpatient Routine Care: A Cross-Sectional Survey. J Med Internet Res.

[15] Chismar W, Wiley-Patton S (2003). Does the extended technology acceptance model apply to physicians. https://ieeexplore.ieee.org/document/1174354.

[16] Kern A, Hong V, Song J, Lipson SK, Eisenberg D (2018). Mental health apps in a college setting: openness, usage, and attitudes. Mhealth.

[17] Torous J, Nicholas J, Larsen ME, Firth J, Christensen H (2018). Clinical review of user engagement with mental health smartphone apps: evidence, theory and improvements. Evid Based Ment Health.

[18] Kaltenthaler E, Sutcliffe P, Parry G, Beverley C, Rees A, Ferriter M (2008). The acceptability to patients of computerized cognitive behaviour therapy for depression: a systematic review. Psychol Med.

[19] Waller R, Gilbody S (2009). Barriers to the uptake of computerized cognitive behavioural therapy: a systematic review of the quantitative and qualitative evidence. Psychol Med.

[20] Gun SY, Titov N, Andrews G (2011). Acceptability of Internet treatment of anxiety and depression. Australas Psychiatry.

[21] Eichenberg C, Wolters C, Brähler E (2013). The internet as a mental health advisor in Germany--results of a national survey. PLoS One.

[22] Koivumäki T, Pekkarinen S, Lappi M, Väisänen J, Juntunen J, Pikkarainen M (2017). Consumer Adoption of Future MyData-Based Preventive eHealth Services: An Acceptance Model and Survey Study. J Med Internet Res.

[23] Jiang LC, Wang Z, Peng T, Zhu JJ (2015). The divided communities of shared concerns: mapping the intellectual structure of e-Health research in social science journals. Int J Med Inform.

[24] Venkatesh V, Morris M, Davis G, Davis F (2003). User Acceptance of Information Technology: Toward a Unified View. MIS Quarterly.

[25] Davis FD (1989). Perceived Usefulness, Perceived Ease of Use, and User Acceptance of Information Technology. MIS Quarterly.

[26] Fishbein M, Ajzen I (1975). Belief, Attitude, Intention and Behavior: An Introduction to Theory and Research.

[27] Fishbein M (1967). Readings in Attitude Theory and Measurement.

[28] Davis FD, Bagozzi RP, Warshaw PR (1992). Extrinsic and Intrinsic Motivation to Use Computers in the Workplace1. J Appl Social Pyschol.

[29] Taylor S, Todd PA (1995). Understanding Information Technology Usage: A Test of Competing Models. Information Systems Research.

[30] Rogers EM (2003). Diffusion of Innovations, 5th ed.

[31] Bandura A (1986). Social foundations of thought and action: A social cognitive theory.

[32] Hoque R, Sorwar G (2017). Understanding factors influencing the adoption of mHealth by the elderly: An extension of the UTAUT model. Int J Med Inform.

[33] Taiwo A, Downe A (2013). The theory of user acceptance and use of technology (UTAUT): a meta-analytic review of empirical findings. Journal of Theoretical and Applied Information Technology.

[34] Liu L, Miguel Cruz A, Rios Rincon A, Buttar V, Ranson Q, Goertzen D (2015). What factors determine therapists' acceptance of new technologies for rehabilitation – a study using the Unified Theory of Acceptance and Use of Technology (UTAUT). Disabil Rehabil.

[35] Hennemann S, Beutel ME, Zwerenz R (2017). Ready for eHealth? Health Professionals' Acceptance and Adoption of eHealth Interventions in Inpatient Routine Care. J Health Commun.

[36] Hennemann S, Witthöft M, Bethge M, Spanier K, Beutel ME, Zwerenz R (2018). Acceptance and barriers to access of occupational e-mental health: cross-sectional findings from a health-risk population of employees. Int Arch Occup Environ Health.

[37] Dünnebeil S, Sunyaev A, Blohm I, Leimeister JM, Krcmar H (2012). Determinants of physicians' technology acceptance for e-health in ambulatory care. Int J Med Inform.

[38] Dohan MS, Tan J (2013). Perceived usefulness and behavioral intention to use consumer-oriented web-based health tools: A meta-analysis.

[39] Zhao Y, Ni Q, Zhou R (2018). What factors influence the mobile health service adoption? A meta-analysis and the moderating role of age. International Journal of Information Management.

[40] Dwivedi YK, Rana NP, Chen H, Williams MD, Nüttgens M, Gadatsch A, Kautz K, Schirmer I, Blinn N (2011). A Meta-analysis of the Unified Theory of Acceptance and Use of Technology (UTAUT). Governance and Sustainability in Information Systems. Managing the Transfer and Diffusion of IT. TDIT 2011. IFIP Advances in Information and Communication Technology, vol 366.

[41] Ebert DD, Berking M, Cuijpers P, Lehr D, Pörtner M, Baumeister H (2015). Increasing the acceptance of internet-based mental health interventions in primary care patients with depressive symptoms. A randomized controlled trial. J Affect Disord.

[42] Schnall R, Higgins T, Brown W, Carballo-Dieguez A, Bakken S (2015). Trust, Perceived Risk, Perceived Ease of Use and Perceived Usefulness as Factors Related to mHealth Technology Use. Stud Health Technol Inform.

[43] Cocosila M, Archer N, Yuan Y (2009). Early Investigation of New Information Technology Acceptance: A Perceived Risk - Motivation Model. CAIS.

[44] Young KS (2005). An empirical examination of client attitudes towards online counseling. Cyberpsychol Behav.

[45] Paramio Pérez G, Almagro BJ, Hernando Gómez A, Aguaded Gómez JI (2015). Validación de la escala eHealth Literacy (eHEALS) en población universitaria española. Rev. Esp. Salud Publica.

[46] Muthén LK, Muthén BO (2012). Mplus User’s Guide. Seventh Edition.

[47] Bentler PM (1990). Comparative fit indexes in structural models. Psychol Bull.

[48] Browne M, Cudeck R (2016). Alternative Ways of Assessing Model Fit. Sociological Methods & Research.

[49] Barak A, Hen L, Boniel-Nissim M, Shapira N (2008). A Comprehensive Review and a Meta-Analysis of the Effectiveness of Internet-Based Psychotherapeutic Interventions. Journal of Technology in Human Services.

[50] Bakker AB, Sanz-Vergel AI, Rodríguez-Muñoz A, Antino M (2019). Ripple Effects of Surface Acting: A Diary Study among Dual-Earner Couples. Span J Psychol.

[51] Ammenwerth E (2019). Technology Acceptance Models in Health Informatics: TAM and UTAUT. Stud Health Technol Inform.

[52] Van Voorhees BW, Hsiung RC, Marko-Holguin M, Houston TK, Fogel J, Lee R, Ford DE (2013). Internal versus external motivation in referral of primary care patients with depression to an internet support group: randomized controlled trial. J Med Internet Res.

